# Comparative Analysis of Gene Expression at Sea Level and High Altitude: A Quantitative Real-Time Polymerase Chain Reaction (qRT-PCR) Approach

**DOI:** 10.7759/cureus.72489

**Published:** 2024-10-27

**Authors:** Siraj B Alharthi, Abdullah H Alsubai, Saad K Almalki, Ahmed M El-Shehawi, Ahmed M Eldebsy, Abdullah A Alsoliman, Rasha F Alharthi, Mohamed Morsi, Dalia Alharthi, Rasha A Mutabaqani

**Affiliations:** 1 Molecular Diagnostic Unit, Al Hada Armed Forces Hospital, Taif, SAU; 2 Biological Sciences, King Abdulaziz University, Jeddah, SAU; 3 Science Department, Shorouq Al Mamlakah International School, Taif, SAU; 4 Department of Biotechnology, Taif University, Taif, SAU

**Keywords:** gene expression, hifa-1, high altitude (ha), hypoxia, mrna expression, physiological adaptation, real-time quantitative pcr (qrt-pcr), sea level (sl), vegf, vhlel

## Abstract

This research studies the gene expression in response to different oxygen environments and looks at high vs low oxygen environments. Tracking down the activity of some of these genes, namely VHLEL, VEGF and HIF-1α, quantitative real-time polymerase chain reaction (PCR) analysis was done for the study group at sea level in Jeddah and at high altitude in Taif city. It has been found that these genes are much more active in higher altitudes which indicates that there is a biological mechanism that makes those specific sites more oversized for the issue of low oxygen. This knowledge is beneficial as it helps in understanding how people grow to live in high-altitude regions. This is positive, especially in justifying the use of the methods in treatment of altitude sickness and other related diseases. Although these findings bring some hope, it would be equally important to include more participants in future studies in order to consolidate our findings and gain deeper understanding of physiological adaptation in low oxygen. This research work has potential significant contribution to the medical profession under conditions of similar environment.

## Introduction

Gene expression, with respect to changes in the environment, is one such cornerstone of molecular biology and genetics that forms the basis of how organisms adapt to changing external conditions [1]. Of these environmental factors, one of the most important is altitude, which through the physiological perturbation of hypoxia or reduced oxygen availability exerts its major influence [2]. Hypoxia is one of the major stressors associated with high altitudes, and it initiates a cascade of cellular and molecular responses that tend to maintain oxygen homeostasis [3]. This research is aimed at the comparison of gene expression at sea level (SL) at the Jeddah coastal area and high altitude (HA), atop the mountainous region in Taif, by means of the three most significant genes, von-Hippel-Lindau (VHL), Vascular Endothelial Growth Factor (VEGF), and hypoxia-inducible factor 1 alpha (HIF-1α), which participate in the critical role of a body’s response in relation to hypoxic stress for maintaining tissue oxygen homeostasis [4,5].

Above 2,500 meters, the amount of atmospheric oxygen becomes noticeably reduced; this can lead to a condition known as hypobaric hypoxia. Physiological adaptation in this respect involves a series of physiological changes. The body enhances oxygen delivery to tissues first by increasing red blood cell production due to erythropoiesis, through the elevation of pulmonary ventilation, and by methods of altering metabolic pathways for maximum oxygen utilization [2].

With chronic exposure to HA, physiological adaptations such as increased capillary density and upregulation of genes that play an important role in oxygen homeostasis important for acclimatization become long-lasting [6]. However, not everyone adapts in the same manner, and maladaptation may underlie the causes of high-altitude illnesses, including high-altitude cerebral edema (HACE) and high-altitude pulmonary edema (HAPE) [3]. The HIF-1α gene is at the very center of the body's molecular response to hypoxia. The gene product, HIF-1α, codes for a subunit of the protein hypoxia-inducible factor 1, a transcription factor that becomes activated in low oxygen conditions and is a key regulator in maintaining oxygen homeostasis [7]. Under normoxic conditions, HIF-1α is rapidly degraded, whereas under hypoxia, it becomes stabilized, accumulates in the cell nucleus, and activates the transcription of genes responsible for angiogenesis, erythropoiesis, and glycolysis [8]. One of the target genes involved in this process is VEGF, or vascular endothelial growth factor, which mediates several aspects of the formation of new vessels-a process termed angiogenesis-to improve oxygen supply in tissues under hypoxic conditions [8].

VHLEL, or von Hippel-Lindau elongin, is a tumor suppressor gene that mediates the normoxic degradation of HIF-1α through tagging for proteasomal degradation [9]. Mutations or changes in VHL can disrupt this pathway, aberrantly stabilizing HIF-1α under conditions of normal oxygen tension; such an anomaly has been implicated in both cancer and maladaptive hypoxic responses [5]. The present study has focused on the differential expression of VHLEL, VEGF, and HIF-1α in subjects dwelling at SL and HA [1]. Considering the fact that these genes are crucial for hypoxic response, their differential expressions are expected to be significantly different between subjects living at sea level and those at high altitude [6,10]. The functions of these types of genes in various oxygen environments may provide an insight into the molecular pathways explaining acclimatization and maladaptation to hypoxia among others, and possible therapeutic targets for altitude-related illnesses [2].

Blood samples from 10 volunteers staying at SL, Jeddah, and HA, Taif, were also collected to answer these questions. Total RNA was prepared using the QIAamp® RNA Blood Mini Kit (Qiagen, Venlo, Netherlands), a reliable method for maintaining RNA integrity from fresh EDTA-treated blood samples [11]. Gene expression was subsequently measured by quantitative real-time polymerase chain reaction (qRT-PCR). qRT-PCR is a sensitive and precise technique that enables the real-time amplification and quantification of messenger RNA (mRNA), hence offering strong data on gene expression across different conditions [11,12].

The results will contribute not only to the explanation of the physiological adaptation of the human body but may also have broader implications for clinical medicine. Hypoxia is considered one of the common denominators for several pathologies including cardiovascular diseases (CVDs), pulmonary hypertension, and even cancer [1,2]. The knowledge gained from such studies may help in the design and formulation of different treatment modalities, particularly those related to hypoxia and high-altitude living. Secondly, the exact genetic markers that are linked to adaptation to hypoxia could, in turn, enable the practice of personalized medicine for individuals living in or traveling to high-altitude areas [4]. The investigation will, therefore, be done in detail on the variously expressed hypoxia-related genes due to varied altitudes. Such a comparison in expression levels of VHLEL, VEGF, and HIF-1α between the populations at SL and HA will be instructive in unraveling the molecular basis of the body's response to hypoxic stress [9]. The outcome of this research work will add to human adaptation in high-altitude environments and further knowledge on hypoxia-related health conditions [2,5].

## Materials and methods

Sample collection

The samples were collected from participants under typical physiological conditions, without any known extremes in their daily habits. All subjects followed normal human routines, with regular and comparable patterns of diet, physical activity, and sleep. Sample collection was performed uniformly across subjects to minimize variability in environmental and lifestyle factors that could influence gene expression. No participants were subjected to unusual physical or environmental stressors during the study. Blood samples were collected from two groups of 10 healthy volunteers, with the aim of comparing and contrasting physiological responses at SL and HA. The samples at sea level were gathered from Jeddah, a coastal city, while the high-altitude samples were collected in Taif, a mountainous region. The partial pressure of oxygen (PO₂) was considered for both study locations. In Taif (1,879 meters above sea level), the average atmospheric pressure is approximately 80 kPa, resulting in a PO₂ of 16.8 kPa. In Jeddah (sea level), the atmospheric pressure is 101.3 kPa, giving a PO₂ of 21.3 kPa [13]. These differences were taken into account when analyzing gene expression data for altitude-sensitive genes like VHL, HIF1a, and VEGF. The primary objective was to compare the differences in gene expression levels of the VHLEL, VEGF, and HIFa-1 genes. qRT-PCR was chosen as it is an efficient, convenient, and accurate method of measuring gene expression by combining traditional RT-PCR with fluorescence resonance energy transfer (FRET) [14].

RNA isolation

RNA was isolated from fresh EDTA-treated whole blood samples. The isolation process was carried out using the QIAamp® RNA Blood Mini Kit, following the manufacturer’s protocol. The procedure involved several steps to ensure the purity and integrity of the RNA, which are critical for accurate qRT-PCR results.

The procedure begins with adding five volumes of Buffer EL to one volume of human blood, then incubating on ice for 10 to 15 minutes followed by brief vortexing twice. Centrifuge for 10 minutes at 4 °C 400 x g, then remove and discard the supernatant entirely. Next is to use two volumes of Buffer EL per volume of whole blood used in step one, quick vortex to resuspend the cells. Then, repeat step two of centrifugation, while adding RLT Buffer to pelleted leukocytes. After that, transfer the lysate directly into a QIAshredder spin column using a 2 ml collection tube, then centrifuge at highest speed for two minutes to ensure homogeneity. Save the homogenized lysate and discard the QIAshredder spin column, then pipette one volume (either 350 μL or 600 μL) of 70% ethanol into the homogenized lysate followed by mixing. Without wetting the rim, carefully pipette sample (including any precipitate that may have developed) into a fresh QIAamp spin column in a 2 mL collection tube. Centrifuge at ≥8000 x g (≥10,000 rpm) for 15 seconds. Place the QIAamp spin column in the supplied fresh 2 mL collection tube. Fill the QIAamp spin column with 700 μL Buffer RW1, then centrifuge to wash for 15 seconds at ≥8000 x g (≥10,000 rpm). Throw away the collecting tube and flow-through. QIAamp spin column should be placed in a fresh 2 ml collection tube. After adding 500 μL of Buffer RPE to the QIAamp spin column, centrifuge at ≥8000 x g (≥10,000 rpm) for 15 seconds. Finally, open the QIAamp spin column carefully, then fill it with 500 μL of Buffer RPE. After closing the cap, centrifuge for three minutes at maximum power (20,000 x g, 14,000 rpm).

qRT-PCR methods and calculations

After completing the procedure above, RNA was isolated using the TRIzol reagent according to its accompanying instructions. This RNA was reverse-transcribed to cDNA with the aid of the MultiScribe RT enzyme kit. The resulting cDNA underwent triplicate analysis through qRT-PCR using Power SYBR Green PCR Master Mix, performed on Applied Biosystems 7500 Real-Time PCR System (Foster City, CA, USA). The threshold cycle (Ct) values of the target genes were compared with those from a reference sample gene to assess relative gene expression alterations. Adjustments for gene expression changes were made by normalizing the expression of the GAPDH housekeeping gene. The normalization of target gene Ct values against GAPDH gene was executed employing the 2-∆∆Ct method described by Livak and Schittgen [15]. Ten samples were selected and sent for qRT-PCR as shown in Table 1 below. 

**Table 1:** This table displays RNA concentration (ng/µl) and absorbance measurements (A260 and A280) for different samples. The ratios of absorbance at 260/280 and 260/230 are used to assess the purity of RNA, with values close to 2.0 indicating good quality RNA. Each sample's measurements are recorded along with the respective sample ID, date, and user name.

#	Sample ID	User name	Date and Time	Nucleic Acid	Unit	A260 (Abs)	A280 (Abs)	260/280	260/230	Sample Type	Factor
5	SAMPLE 1 D	ccis	7/6/2023 10:05:57 AM	56.7	ng/µl	1.417	0.682	2.08	1.28	RNA	40
7	SAMPLE 2 D	ccis	7/6/2023 10:07:26 AM	71.2	ng/µl	1.78	0.851	2.09	1.59	RNA	40
9	SAMPLE 3 D	ccis	7/6/2023 10:08:18 AM	49.3	ng/µl	1.232	0.581	2.12	0.92	RNA	40
11	SAMPLE 4 D	ccis	7/6/2023 10:09:10 AM	87.4	ng/µl	2.184	1.037	2.11	1.94	RNA	40
13	SAMPLE 5 D	ccis	7/6/2023 10:10:17 AM	70.1	ng/µl	1.753	0.84	2.09	2.01	RNA	40
2	BLANK	ccis	7/9/2023 10:38:36 AM	-0.3	ng/µl	-0.007	-0.01	0.65	0.65	RNA	40
6	SAMPLE 8D	ccis	7/9/2023 10:44:38 AM	56.6	ng/µl	1.416	0.675	2.1	0.9	RNA	40
7	SAMPLE 9	ccis	7/9/2023 10:45:21 AM	42.4	ng/µl	1.059	0.502	2.11	1.53	RNA	40
10	SAMPLE 10D	ccis	7/9/2023 10:48:54 AM	64.1	ng/µl	1.602	0.78	2.05	0.79	RNA	40
11	SAMPLE 11	ccis	7/9/2023 10:50:13 AM	46.9	ng/µl	1.172	0.58	2.02	0.46	RNA	40
13	SAMPLE 12	ccis	7/9/2023 10:52:23 AM	49.6	ng/µl	1.24	0.605	2.05	1.29	RNA	40

Gene expression analysis

In this study, the gene expression levels were analyzed using the ΔΔCt method, the most accepted quantitative technique in RT-PCR analysis [2]. The Ct values (threshold values) of the target genes were first normalized against the Ct values of the housekeeping gene GAPDH. GAPDH is chosen due to its stability under experimental conditions, leading to accurate normalization [16].

Differences in Ct values between the target gene and GAPDH (i.e. ΔCt) were calculated for both samples (SL and HA). This ΔCt value normalized each gene of interest to the housekeeping gene (GAPDH). Then, we calculated ΔΔCt by subtracting the ΔCt of sample (HA) from the ΔCt of the control (SL). To get a comprehensive number, we calculate for 2^-ΔΔCt, which gives us the fold change of expression. With this value we are able to determine whether a certain gene has been upregulated or downregulated. Values greater than 1 indicate upregulation of target compared to control, while values less than 1 indicate downregulation. 

Primer sequences

To maintain accurate and specific amplification of our target genes, certain primers were used for the qRT-PCR analysis. These primers were designed to have high specificity and efficiency, as indicated by their performance in preliminary experiments. These primers targeted the genes GAPDH, VHLEL, VEGF, and HIFa-1. The primer sequences, along with their respective accession numbers and amplification efficiencies, are listed in Table 2. An ideal primer efficiency of 100% implies that the targeted DNA exactly doubles after each PCR cycle. 

**Table 2:** This table shows the primers used for qRT-PCR amplification with their respective gene names, primer sequences, accession numbers, efficiencies, and melting temperatures

No.	Gene	Primer sequence (5'-3')	Accession number	Efficiency %	Tm
1	GAPDH	F:CAAGGTCATCCATGACAACTTTG R:GTCCACCACCCTG TTGCTGTAG	NM_017008.4	99.64	-
2	VHLEL	F: GAGATGCAGGGACACACGAT R: AGGCAGACAAGTCACCAACC	NM_000551.4	94.14	60 60
3	VEGF	F: TCCTGGAGCGTGTACGTTG R: ACATTCCCCTCCCAACTCAAG	NM_001171623.2	95.24	58 53
4	HIFa-1	F: TTTCCTCAGTCGACACAGCC R: GTGCAGGGTCAGCACTACTT	BC012527.2	91.47	60 60

GAPDH was used as the housekeeping gene in this study due to its stable expression across the samples. The forward and reverse primer sequences for GAPDH, listed in the table, were optimized to yield an amplification efficiency of 99.64%, indicating highly efficient primer binding and amplification.

VHLEL had an amplification efficiency of 94.14%, with a melting temperature (Tm) of 60°C for both the forward and reverse primers. This gene plays a role in cellular oxygen-sensing pathways, and its expression was measured to investigate its potential involvement in the experimental conditions.

VEGF, an important regulator of angiogenesis, exhibited an amplification efficiency of 95.24%, with forward and reverse primer Tm values of 58°C and 53°C, respectively. The primer efficiency for VEGF was nearly optimal, ensuring accurate quantification of this gene's expression, which is critical in studies of hypoxia and tissue vascularization.

HIF1α, a transcription factor activated under low oxygen conditions, had an efficiency of 91.47%, with both primers having a Tm of 60°C. This gene was of particular interest due to its central role in the cellular response to hypoxia, making it a key target for understanding how cells adapt to oxygen deprivation.

The high amplification efficiencies listed for each gene indicate that the primers were well-designed and functioned efficiently during qRT-PCR, ensuring reliable quantification of gene expression. The Tms were carefully chosen to ensure specific binding of the primers during the annealing phase of PCR, thereby reducing non-specific amplification.

Data analysis

This study employed both absolute and relative quantification methods to analyze the qRT-PCR data. Absolute quantification determines the input copy number by relating the PCR signal to a standard curve. Relative quantification, used in this study, relates the PCR signal of the target transcript in the HA group to that of the SL group, serving as an untreated control.

## Results

The study’s quantitative analysis revealed significant alterations in mRNA expression levels of the genes VHLEL, VEGF, and HIFa-1 when comparing samples from HA to those from SL. The qRT-PCR data presented below demonstrate these changes.

VHLEL gene expression

**Figure 1:**
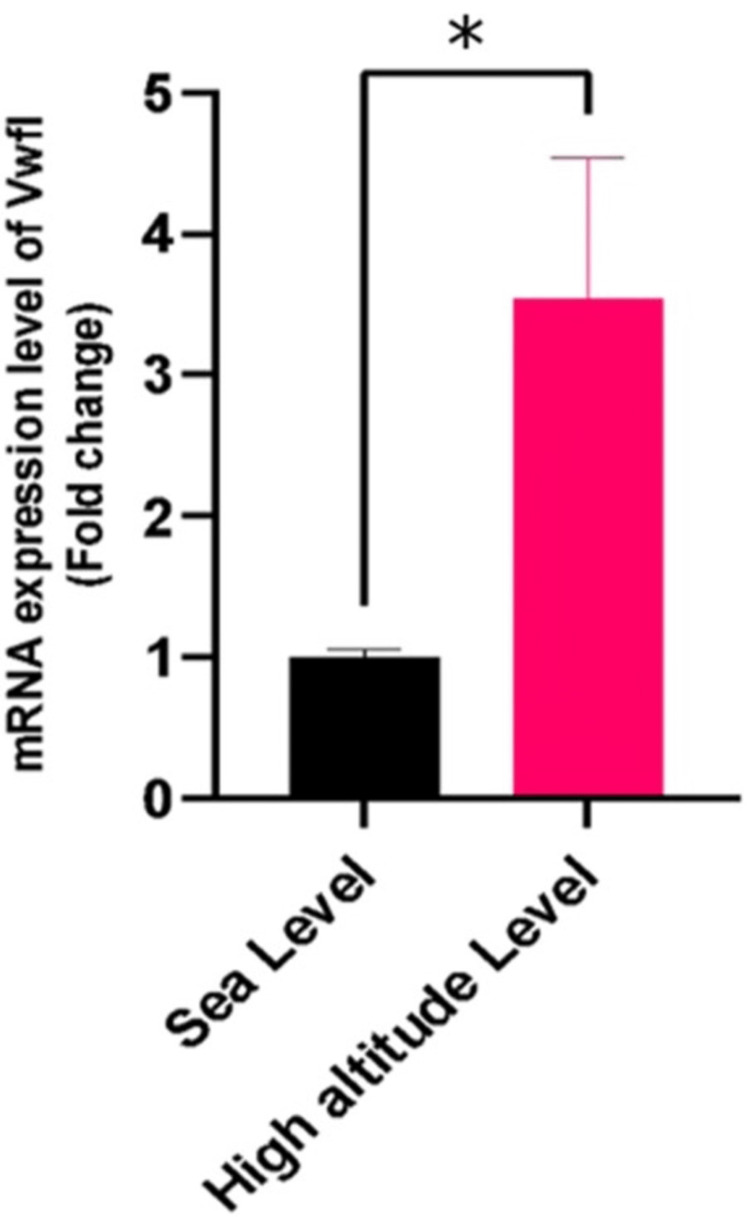
VHLEL mRNA Expression in Sea Level (SL) vs. High Altitude (HA) Samples * Indicates significant results (p < 0.05).

Figure 1 presents a comparision of VHLEL mRNA expression levels between the SL and HA groups. The data reveals a significant upregulation of VHLEL mRNA expression in the HA group as compared to the SL as control. A fold change of 3.5 (p<0.05) implies that VHLEL expression is almost quadrupled during hypoxic conditions.

VEGF gene expression

**Figure 2:**
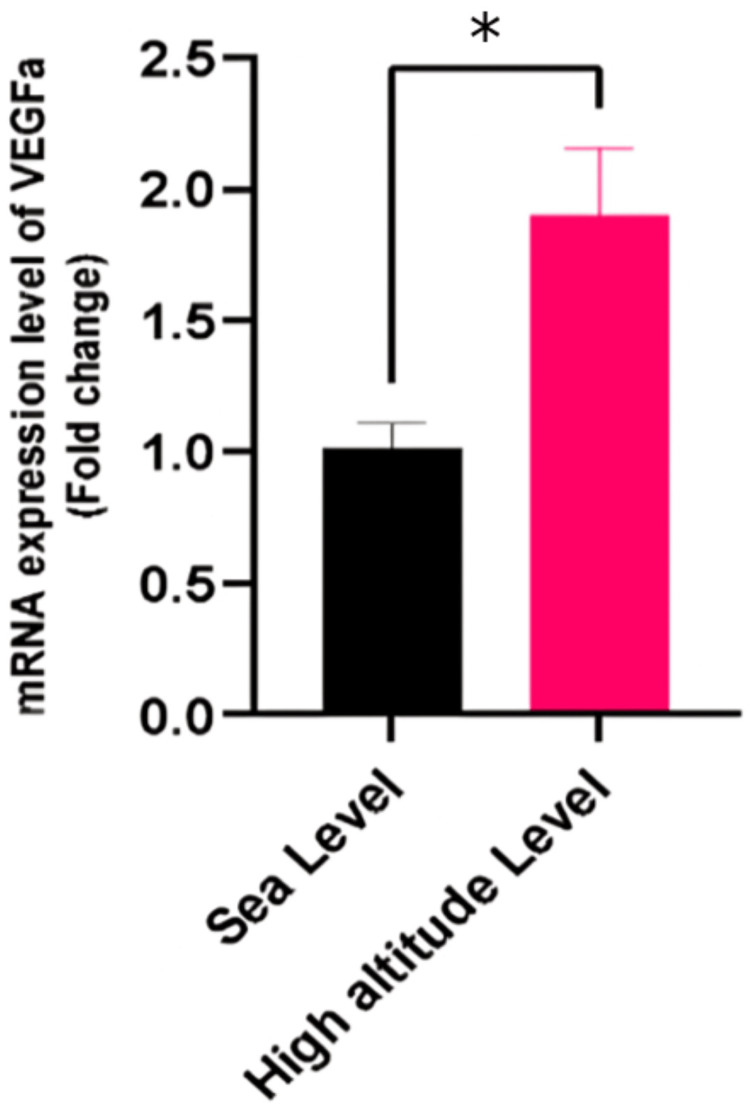
Comparative Analysis of VEGFa mRNA Expression in Sea Level (SL) vs. High Altitude (HA) Samples * Indicates significant results (p < 0.05).

As depicted in Figure 2, the VEGF gene also showed a notable increase in mRNA expression in the HA group, with a fold change of 1.9 (p<0.05). VEGF is a critical factor in angiogenesis, and its upregulation may indicate a physiological adaptation to ensure adequate oxygen supply under reduced atmospheric oxygen levels. VEGF expression is almost doubled during hypoxic conditions.

HIFa-1 gene expression

**Figure 3:**
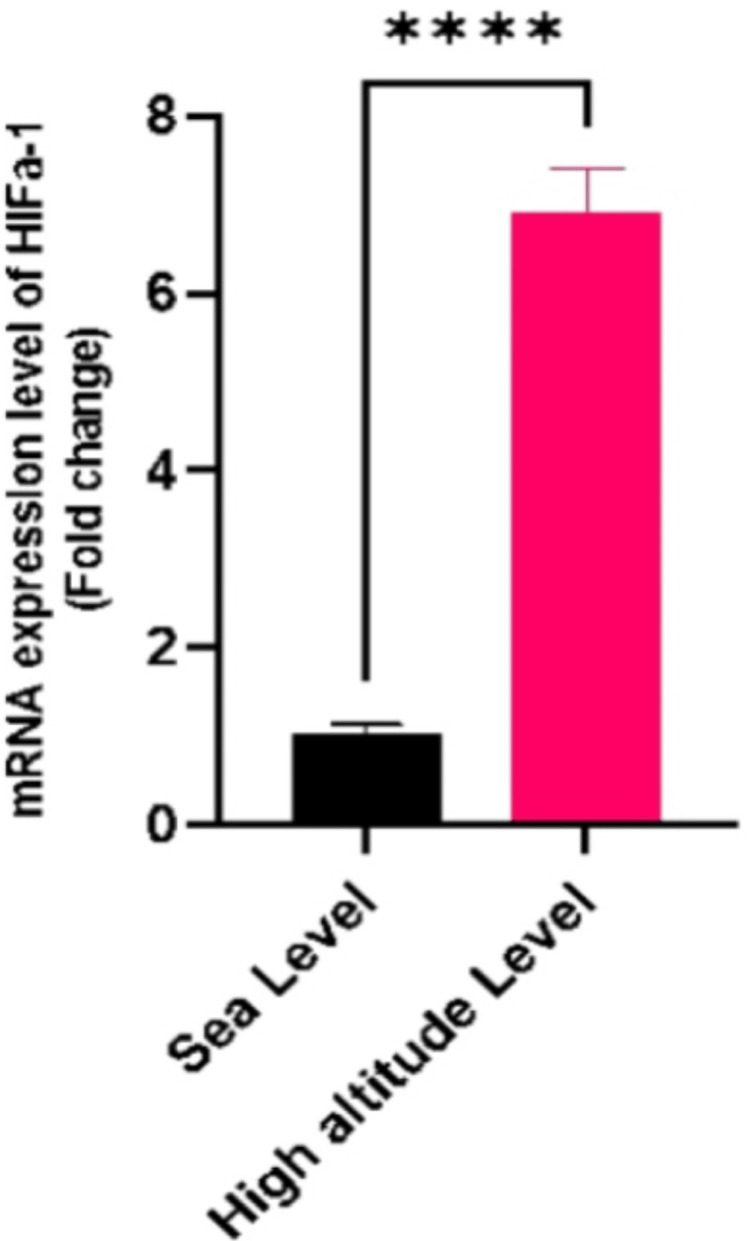
HIFa-1 mRNA Expression in Sea Level (SL) vs. High Altitude (HA) Samples **** indicates extremely significant results (p < 0.0001).

The most pronounced change was observed in the HIFa-1 gene, where Figure 3 shows a 6.9-fold increase in mRNA expression in the HA group compared to the SL group (p<0.05). HIFa-1 is a key regulator in the cellular response to hypoxia, and its substantial upregulation aligns with the body’s adaptive mechanisms to HA environments [17]. HIFa-1 shows a profound near-sevenfold increase in expression.

Statistical analysis

To determine the statistical significance of the observed differences, a Student’s T-test was employed. In our analysis we used a p-value threshold of 0.05, corresponding to the widely accepted significance level of 5%, representing the probability of Type 1 error (α). Fold change was calculated using the 2^-ΔΔCt method.

## Discussion

Adaptation of human populations to diverse environmental conditions, particularly at high altitudes, has been a critical focus in genetic and physiological research. This study offers a comprehensive analysis of genetic variations tied to high-altitude adaptation in different regions of West Saudi Arabia, honing in on crucial genes like HIF-1α, VEGF, and VHL, which play key roles in regulating the body’s response to hypoxia-a hallmark of high-altitude environments [3,7].

A distinct pattern of variation was found in the HIF-1α gene, a well-known regulator of oxygen balance, among HA individuals. This suggests a unique cellular response to hypoxia. Previous research has shown that HIF-1α has a vital role in helping cells adjust to low oxygen conditions. In high-altitude populations, variants in this gene help enhance oxygen delivery and efficiency in low-oxygen settings, as seen in Tibetan communities [18,4].

The VEGFa gene, essential for angiogenesis and vascular permeability, also exhibited significant differences in the HA group. This indicates that people living at high altitudes may have a greater capacity to form new blood vessels, aiding in oxygen distribution to tissues [8]. Similar adaptations have been observed in other high-altitude populations, such as the Andeans, where increased vascularization helps counter the low oxygen levels [2].

In the HA group, single nucleotide polymorphisms (SNPs) were prominent, linking VHL to high-altitude adaptation through its role in controlling the breakdown of HIF proteins. Variants of VHL found in Tibetans assist in optimizing the hypoxia response by managing HIF levels, reducing the risk of erythrocytosis, a common altitude-related disorder [12]. By regulating HIF-1α, the VHL gene ensures a balanced cellular response to hypoxia, preventing excessive blood vessel formation or harmful metabolic shifts [10].

Phylogenetic analysis further reinforced these genetic observations. Individuals from the HA group, particularly those with SNPs in the VHL, HIF-1α, and VEGFa genes, clustered together, reflecting a shared evolutionary background. This clustering suggests that selective pressures from high-altitude environments drive similar evolutionary strategies, echoing those seen in other populations like the Tibetans and Andeans [10]. The study not only broadens our understanding of how high-altitude populations adapt but also strengthens the case for region-specific genetic markers for altitude adaptation [1].

Beyond genetic variations, transcriptomic and proteomic studies shed more light on how organisms adapt to hypoxia at high altitudes. Transcriptomic data from high-altitude vertebrates point to differences in genes regulating oxygen transport and metabolism. Meanwhile, proteomic analysis of human platelets exposed to low oxygen levels revealed changes in proteins central to oxidative phosphorylation and the electron transport chain, showing enhanced mitochondrial activity as a compensatory mechanism [19]. These changes reflect molecular fine-tuning within a larger systemic adaptation to high-altitude environments, highlighting the intricate interplay between genetic and proteomic elements in coping with hypoxia.

The study’s findings are pivotal for understanding how humans adjust to the hypoxia associated with high altitudes, with important medical implications. Identifying specific genetic adaptations among high-altitude residents of Taif, Saudi Arabia, could offer protection against altitude-related illnesses such as pulmonary edema and chronic mountain sickness. The regional SNPs in HIF-1α, VEGFa, and VHL provide a clear path for future research into therapies targeting the hypoxia response in altitude-related conditions [20].

Study limitations

This study is limited by the small sample size of only 10 volunteers from each group, which may restrict the generalizability of the findings. The limited number of participants may not adequately capture the variability in mRNA expression across different genes in response to high altitude and sea level conditions. Further research with a larger amount of volunteers is needed to validate these results and enhance the statistical power of the analysis.

## Conclusions

The present study has emphasized that high-altitude residence has an influence on differential gene expression, especially upregulating VHLEL, VEGF, and HIFa-1 genes, which combinedly provided a molecular basis for physiological adaptations required for sustaining homeostasis under hypoxic conditions. This increased expression of these genes reflects not only immediate responses to hypoxic stress but also suggests long-term adaptations that could influence disease susceptibility, including conditions such as pulmonary hypertension and certain cancers. Moreover, potential therapies based on hypoxia-responsive genes could be designed for treatments or adaptations with patients who have chronic hypoxia or must acclimatize to high-altitude conditions. Our research adds to the global understanding of high-altitude adaptation by pinpointing specific genetic markers in the population of West Saudi Arabia, which shows parallels with adaptations observed in Tibetan and Andean populations. These cross-population comparisons are crucial for developing a comprehensive model of human adaptation to diverse environmental stresses, providing insights that could benefit not only local but also global strategies for managing altitude-related health issues. It is such studies, with an extended range of genes and in populations that are more genetically diverse, which will have the key to a comprehensive explanation of the various interactions between the gene expression systems and environmental signals and the potential for new treatment modalities for diseases related to hypoxia.
